# The first reported case of factor V Leiden mutation with agenesis of superior vena cava: A case report

**DOI:** 10.1097/MD.0000000000010511

**Published:** 2018-06-01

**Authors:** Valter Romão De Souza, Victor Moreira Valença de Lemos, Daniell de Siqueira Araújo Lafayette, Roberto Souza de Lemos, Luciana de Barros Correia Fontes, Glaydes Maria Torres de Lima, Cláudia Wanderley de Barros Correia, Ana Lucia Coutinho Domingues, Amanda Queiroz da Mota Silveira Aroucha, Igor Macedo de Oliveira, Gabriel Pinheiro Santos, Ana Maria Vanderlei, Vladmyr Moreira Valença de Lemos, João Vitor Sostenes Peter, Juliana Oliveira Vieira, Aline Rayane Pereira Mariano, Turíbio Anacleto Gomes, Alice Marcelle de Souza Ferraz, José Henrique Sousa do Amaral, Esdras Marques Lins, Marcos André Cavalcanti Bezerra, Paulo Sérgio Ramos de Araújo, Adriana Ferraz de Vasconcelos, Maria da Conceição de Barros Correia, Manuela Freire Hazin Costa

**Affiliations:** a Universidade Federal de Pernambuco, Recife, Brazil; b Fundação de Hematologia e Hemoterapia de Pernambuco, Recife, Brazil; c Federal University of Pernambuco Society for Haematology, Recife, Brazil; d Instituto de Medicina Integral Prof. Fernando Figueira (IMIP), Recife, Brazil; e Universidade de Pernambuco, Av. Agamenon Magalhaes, Recife, Brazil.

**Keywords:** agenesis, cardiovascular abnormalities, deep vein thrombosis, factor V Leiden mutation, superior vena cava

## Abstract

**Rationale:**

Total absence of superior vena cava (ASVC) is a very rare anomaly, and the patient usually suffers from superior vena cava syndrome (SVCS) or conduction disturbances.

**Patient concerns:**

We report a 36-year-old white male, born and living in Brazil, without comorbidities presented to hematologist thrombotic episodes even under anticoagulant therapy. On his first hematologic appointment, he had no active complaints except by the fullness after meals, and his physical examination presented remarkable collateral circulation in the chest.

**Diagnoses:**

Congenital ASVC associated with factor V Leiden mutation.

**Outcomes:**

In his magnetic resonance angiography of the thorax, a great amount of collateral circulation and communication of the azygos and hemiazygos veins with inferior vena cava were evident, as well as the absence of the upper cava vein. Furthermore, heterozygous genetic mutation was found for Leiden factor V.

**Lessons:**

This case gives us the lesson that we need to include ASVC in the differential diagnosis of SVCS. The importance of the V-Leiden factor as a joint risk with this congenital defect for venous thromboembolism episodes was also highlighted.

## 1 Introduction

The superior vena cava (SVC) drains blood from the head and upper extremities to the heart. When SVC blood flow becomes limited, whether by extraluminal compression, congenital defects in blood vessel formation, or intraluminal obstruction, superior vena cava syndrome (SVCS) can occur. As SVC obstruction progresses, blood and pressure in the venous system increases and the collateral vessels dilate. The severity of the symptoms is related to the speed and degree of this blockage.^[1]^

Inherited anomalies of SVC are caused by variations in the embryonic development of the thoracic venous system. Due to the complexity of its developmental stages, the cardinal vein system can undergo a variety of congenital anomalies during the embryonic period. During the fourth week of gestation, 2 large symmetric systems of cardinal veins, right and left precursor veins, drain the upper portion of the embryo. At the eighth week, the right and left anterior cardinal veins become connected by an anastomotic duct (Marshall ligament), which later becomes the SVC on the right side and the left brachiocephalic vein on the left side. However, when this formation fails and cannot be obliterated, the congenital problems in the formation of the SVC develop.^[2–4]^

The absence of superior vena cava (ASVC) is a very rare congenital variant of the disorders of the venous tributary vein, with few cases recorded.^[5]^ It is usually asymptomatic and commonly detected incidentally during thoracic investigation by computed tomography (CT) scan or even necropsy findings.^[6]^ In this context, there is no description, in the investigated literature, of a case including congenital SVC agenesis combined with Leiden Factor V mutation, object of the present case report.

## 2 Case presentation

A 36-year-old white male, born and living in Brazil, attended the hematological outpatient clinic of the Hospital das Clínicas (HC), a hematologic reference hospital located in the state of Pernambuco, northeast Brazil in 2015. He was referred to the Medical Clinic Sector of HC to investigate the repeated episodes of deep venous thrombosis (DVT) even under the effect of anticoagulant drugs. He reported having presented frequent dyspnea on medium exertion after meals 10 years ago. In 2010, he developed DVT in lower limbs with no alteration on activated partial thromboplastin time (aPTT). Doppler ultrasonography showed affecting the popliteal vein extending to the external iliac. The chronic use of Marevam (4 mg/day) was prescribed after DVT episode. In the medical history, there was a record of previous hospitalizations with worsening of the clinical picture of SVCS, right hepatic vein thrombosis, and hepatic inferior vena cava and thrombosis of the upper limbs. The patient also had a history of alcohol consumption, regularly at weekends (2–4 glasses of beer), from 16 to 28 years of age, reporting abstinence for the last 8 years. He reported neither smoking nor making use of illicit drugs. From family history, his father suffers from systemic arterial hypertension (SAH) and his brother was diagnosed with depression.

**Figure 1:**
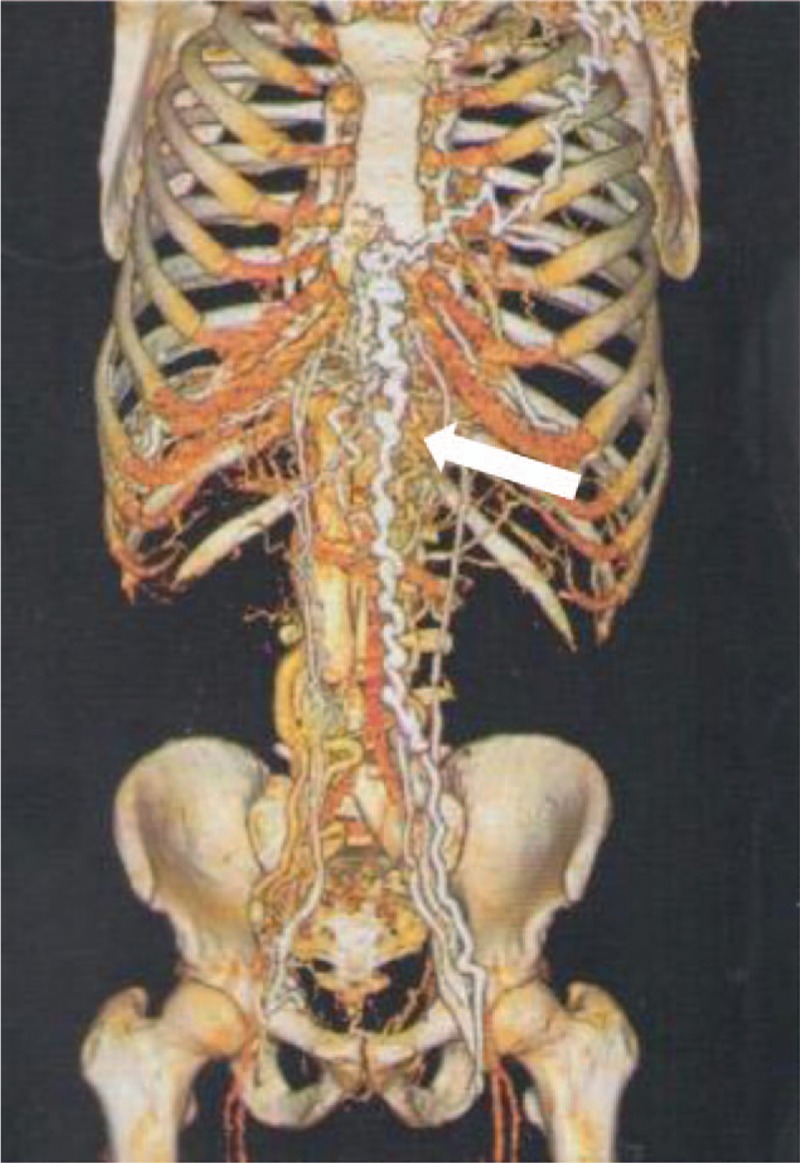
Three-dimensional restructuring image of CT revealed collateral circulation in the chest.

**Figure 2:**
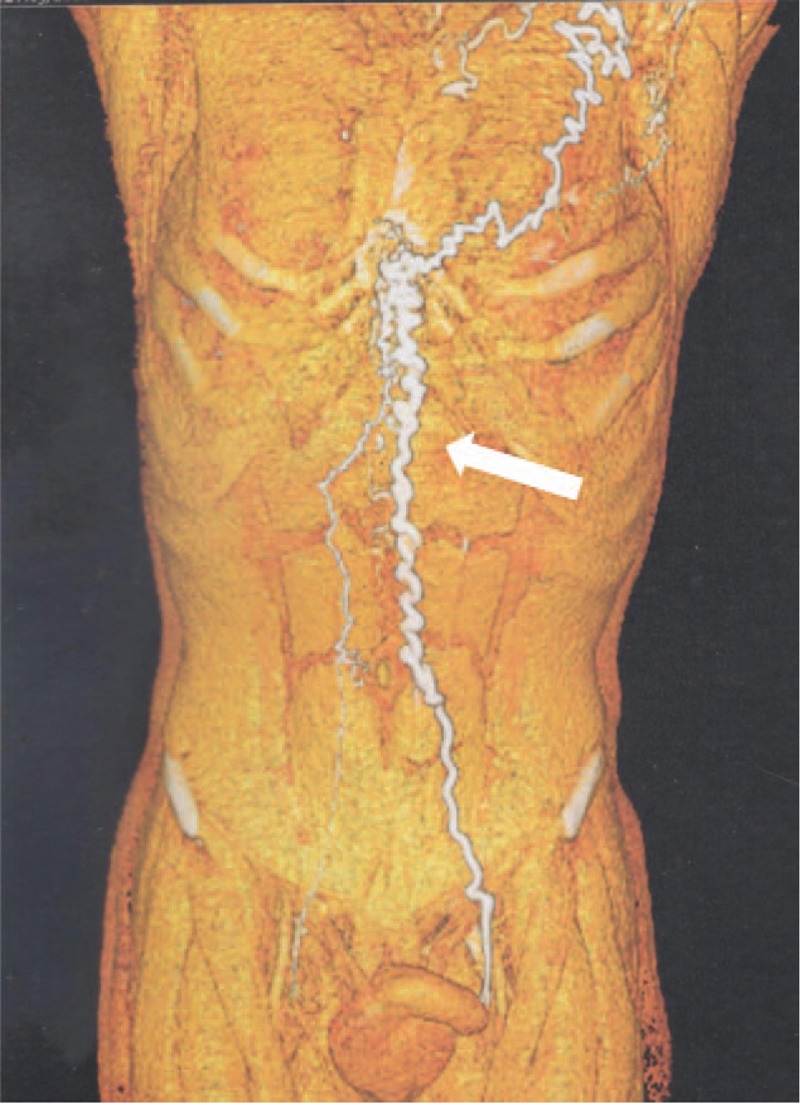
Three-dimensional restructuring image of CT revealed collateral circulation in the chest.

**Figure 3:**
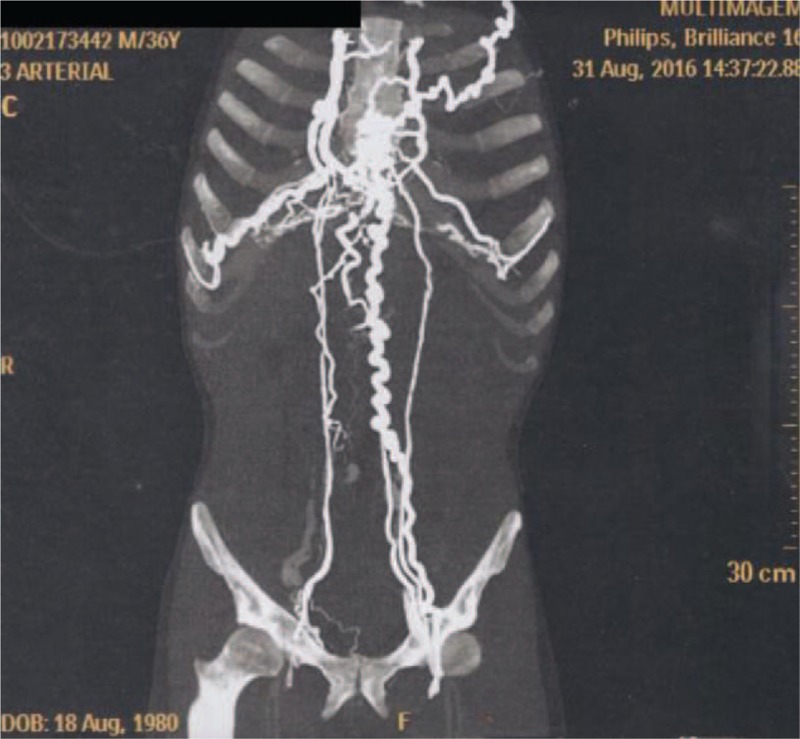
Magnetic resonance angiography of the thorax showing remarkable collateral circulation in the chest.

**Figure 4:**
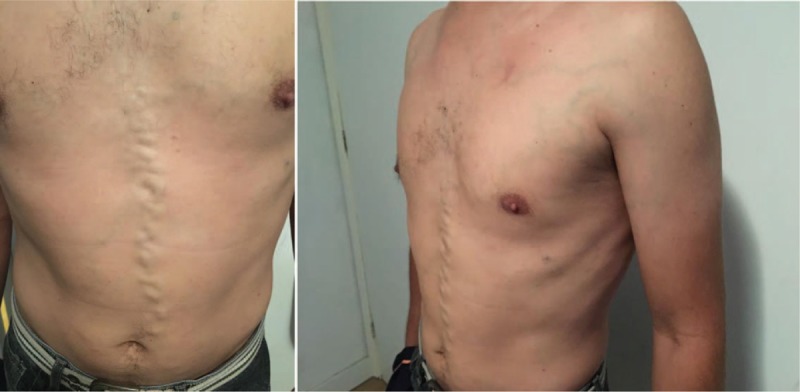
Collateral circulation in the chest.

On physical examination, there was a remarkable collateral circulation in the chest (Figs. 1–4). Laboratory tests did not show alterations, except for aPTT of 42 seconds (normal: 28–32 seconds). Lupus anticoagulant, anti-�2glycoprotein-1, and anticardiolipin antibodies were within normal parameters. Furthermore, tumor markers levels (alpha-fetoprotein, beta-2-microglobulin, beta-human chorionic gonadotropin, C-kit/CD117, calcium, Ce A, and prostate-specific antigen) and thyroid/parathyroid ultrasonography were within normality, which did not suggest a malignancy that could result in the obliteration of the SVC and thrombotic events.

**Figure 5:**
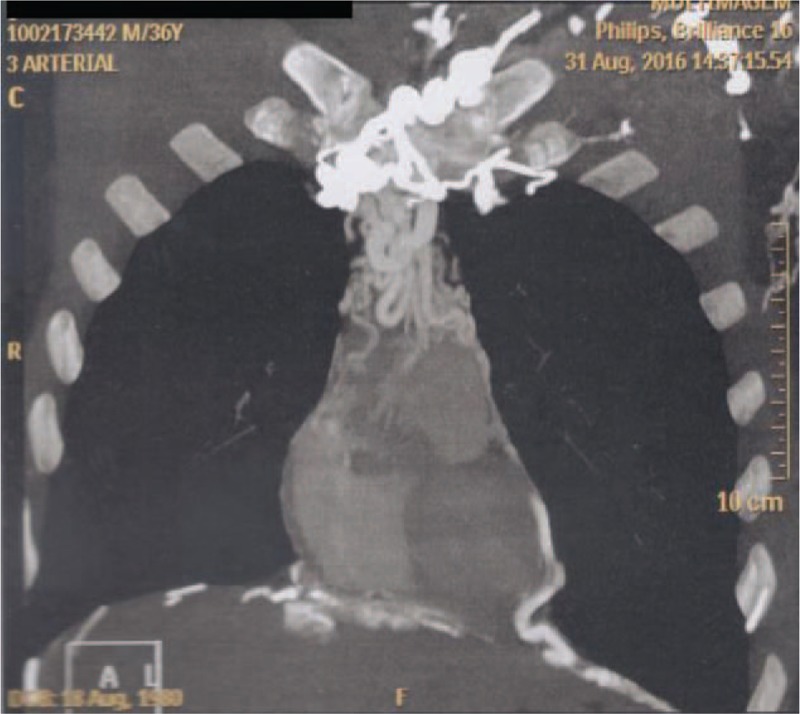
Magnetic resonance angiography of the thorax showing absence of upper vena cava.

**Figure 6:**
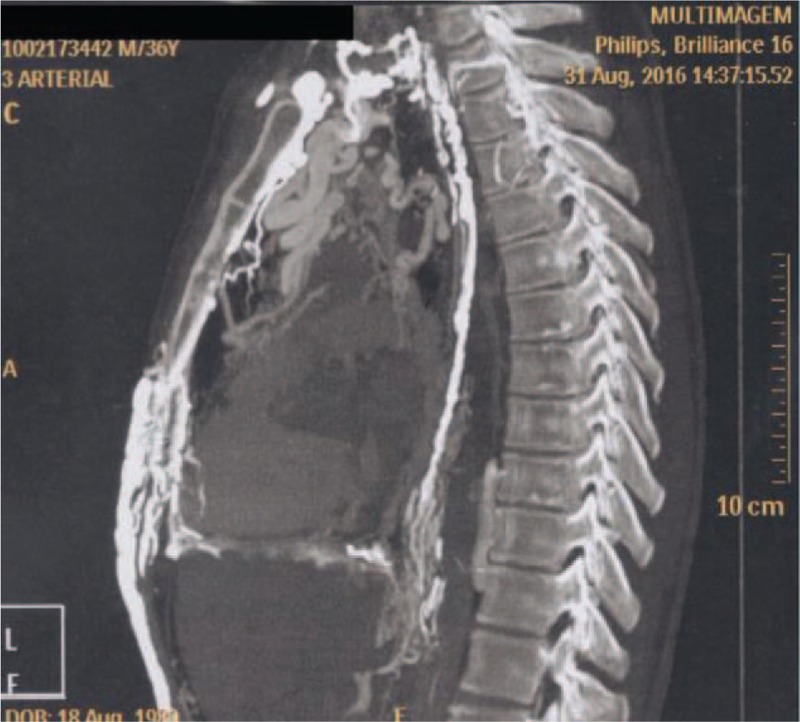
Magnetic resonance angiography of the thorax showing absence of upper vena cava.

**Figure 7:**
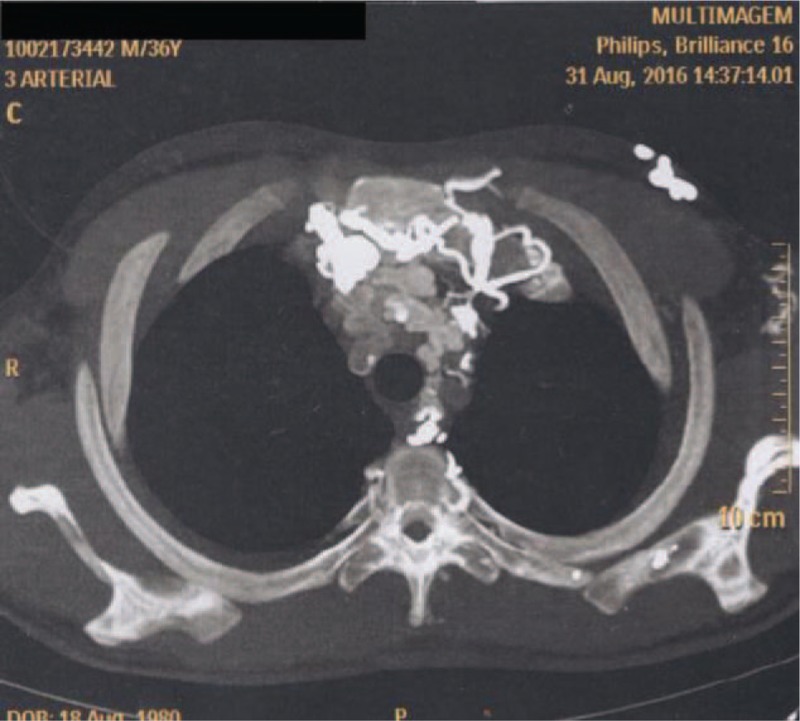
Magnetic resonance angiography of the thorax.

**Figure 8:**
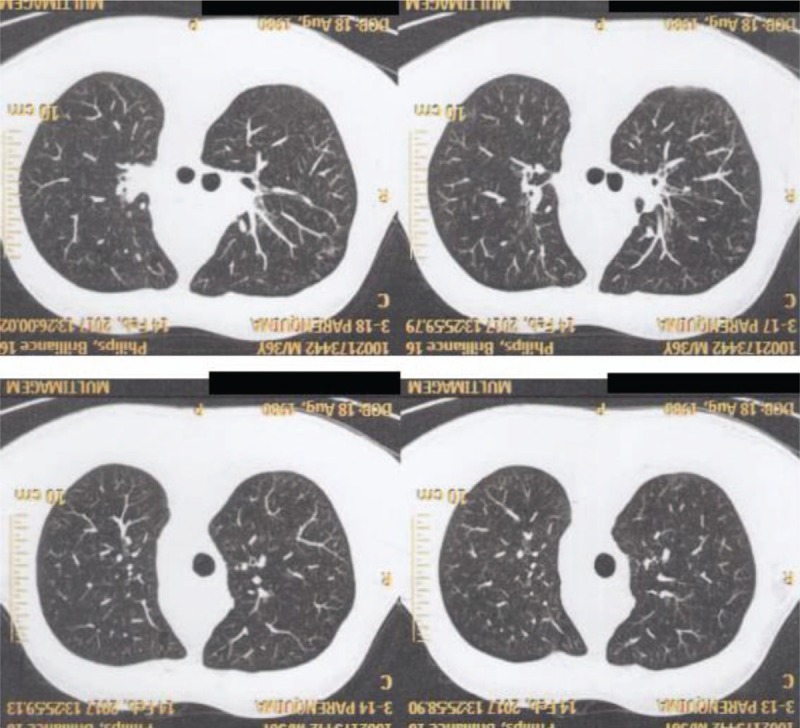
Magnetic resonance angiography of the thorax.

**Figure 9:**
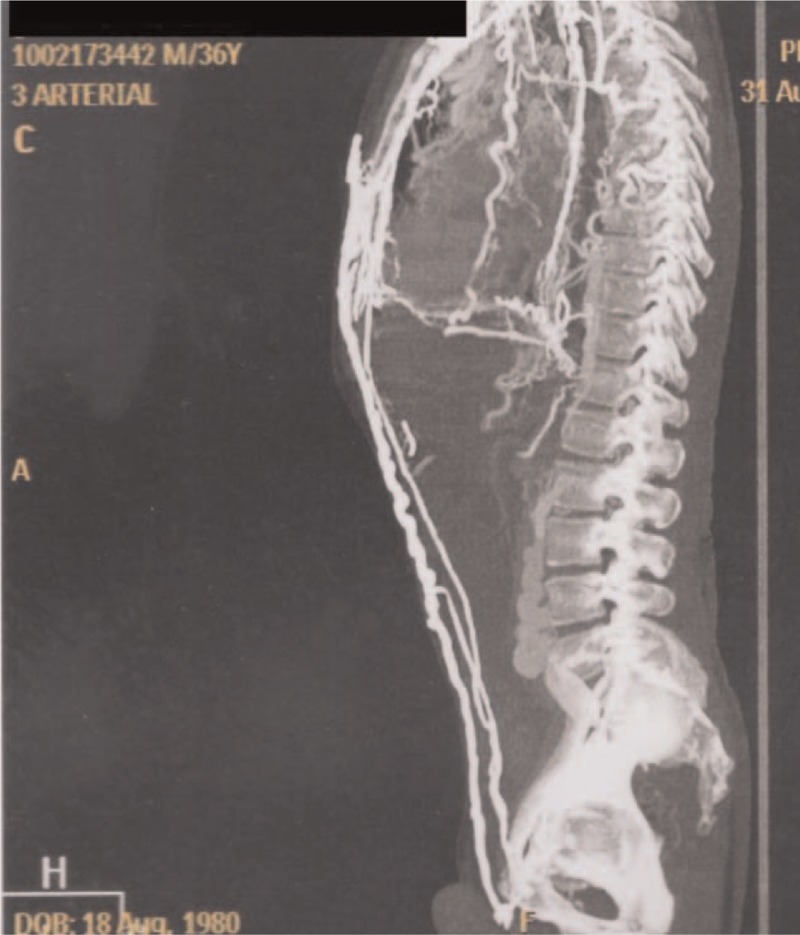
Magnetic resonance angiography of the thorax showing absence of upper vena cava.

Heterozygous genetic mutation was found for Leiden factor V, but no genetic mutation for nocturnal paroxysmal hemoglobinuria, another differential diagnosis for hypercoagulability. Upper endoscopy revealed multiple esophageal varices. Abnormalities were observed in the combined thoracic-abdominal-pelvic CT, which presented congenital absence of SVC and nonexistent arch of azygos (Figs. 5–7). In the angiotomography of the thorax, a great amount of collateral circulation and communication of the azygos and hemiazygos veins with inferior vena cava were evidenced, due to the absence of SVC (Figs. 8 and 9).

In accordance with the pathological alterations that characterize the hypercoagulability states, the protocol for the prevention of the formation or recurrence of thrombosis using vitamin K antagonists was then considered. In 2016, the patient was treated with a combination of enoxaparin (60 mg/day) and warfarin (5 mg/day) and continued with monthly consultations at the hematology clinic previously mentioned.

In April 2017, he was hospitalized in the same medical service due to severe abdominal pain in the upper right quadrant, lower limb edema, progressive and continuous dyspnea reported a month ago.

The new physical examination revealed soft petechial palate, jugular distention, absent breathing sounds, and impaired resonance percussion at the right base. CT showed right pleural effusion and thrombosis of the hepatic vein. The patient's family refused consent for cardiopulmonary resuscitation when he presented cardiorespiratory arrest, evolving to death.

### 2.1 Review of literature and discussion

Early diagnosis of inherited SVC formation problems is difficult because most patients are asymptomatic, requiring the use of imaging such as magnetic resonance angiography.^[7]^ Nevertheless, clinical manifestations of SVCS such as jugular distention, dyspnea, and collateral circulation in the chest wall can be observed, as reported by the present case. The appearance of collateral circulation depends on the degree of SVC obstruction, being evident in the complete absence of SVC or when the subcutaneous veins are pronounced, with varicose appearance on the anterior wall of the thorax. Telangiectasias were also observed in this patient.^[8]^

The azygos, internal thoracic or mammary veins, lateral thoracic veins, as well as the paraspinal veins and the periesophagous venous plexus engorge in an attempt to find a way of returning blood from the upper extremities to the heart. The pressure in these vessels rises because the venous drainage is impaired. This anomaly can cause slow return of blood through the collaterals, leading to increased venous pressure, venous stasis, and thrombus formation. The patient showed prominent development of collateral vessels, which could have begun to develop in the fetal period, allowing sufficient venous return and making the patient asymptomatic for a given moment, even in the absence of the SVC.^[4]^

As for the presence of the Leiden Factor V, this mutation is the most important genetic risk factor for thrombosis and the most common cause of genetically inherited thrombophilia in the general population, consisting of a hereditary, autosomal dominant alteration, which interferes with protein C performance.^[9]^ The exchange of the factor V 50 (factor V Leiden) by a glutamine (R506Q) induces the resistance of activated protein C. In this way, the cleavage and inactivation of the factor Va is done in an unsatisfactory manner, leading to its accumulation and, consequently, increased risk of thrombosis.^[9,10]^ In the present case report, the patient was heterozygous, which would not justify severe thrombosis attacks, leading to the interpretation that the association of heterozygous Leiden V-factor with congenital defect of the SVC may intensify thrombotic events. This congenital anomaly suggests that hypercoagulable state created results of physiologic stress.^[11]^

The diagnosis of Leiden Factor V can be performed indirectly by functional assays of activated protein C resistance or by directly analyzing the DNA as performed in the present case. Genomic DNA was extracted from peripheral blood of the patient. Amplification of exon 10 of the factor V gene was done by polymerase chain reaction in a mixture of 54 mM Tris-HC1, pH8.8, 5.4 mM MgCl, 5.4 μM EDTA, 13.3 mM (NH_4_)_2_SO_4_, 10% DMSO, 8 mM βmercaptoethanol, 0.4 mg/mL BSA, 0.8 Mm of each nucleoside triphosphate, and 400 ng of each primers: sense (5’-CTTGAAGGAAATGCCCCATTA-3’) and antisense 5’-TGCCCAGTGCTTAACAAGACCA-3’), 500 ng of genomic DNA, and 2 U Taq polymerase. The reaction involved 30 cycles of incubation at 94°C (40”), 57°C (40”), and 72°C (2’). A fragment of 220 bp was obtained and 5–10 p1 of PCR product was digested with 0.5 U Mnll. This region of exon 10 of the factor V gene contains 2 restriction sites for MnlI at nucleotides 1637 and 1694. After digestion of the normal FV gene (allele 1691G), fragments of 116, 67, and 37 bp were observed in a 2% agarose gel. When allele 1691A was present, the cleavage site for MnlI at nucleotide 1694 was involved and fragments of 67 and 153 bp were found. Using this approach for our patient, the latter of FV Leiden G1691A was positive for its heterozygous variation that presented 4 fragments (37 bp, 67 bp, 116 bp, 153 bp) generated by the loss of restriction of one of the alleles. All other molecular analyses (MTHFR C677T, PT G20210A) were determined by restriction fragment length polymorphism polymerase chain reaction, as previously described.

Regarding increased aPTT of the patient, it can be explained because the patient was under anticoagulation. aPTT test before the use of anticoagulation therapy was normal. Nevertheless, we investigated the antiphospholipid syndrome (aPS) diagnosis due to our patient presenting uncommon thrombotic events for his age range but his apt was abnormal. The patient did not fulfill diagnostic criteria Sapporo or Sydney criteria (2006); the aPS is characterized by a history of repeated venous or arterial thrombosis and/or fetal losses associated with the presence of persistently elevated seric antiphospholipid antibodies (aPl), mainly anticardiolipin, lupus anticoagulant, and anti-β2 glycoprotein, measured in 2 different occasions at least 12 weeks apart.^[12]^

Considering the rarity of the disease, there is no strong evidence-based approach to prove efficacy, given the particularity found. Treatment was directed at preventing the formation or recurrence of thrombosis. Vitamin K antagonist throughout life is necessary for patients with this pathology. Surgical correction could be an option for management, depending on the severity of venous stasis symptoms and the response to conservative medical treatment.

## 3 Conclusion

The report of this case reinforces the need to include ASVC in the differential diagnosis of SVCS. The importance of the V-Leiden factor as a joint risk with this congenital defect for venous thromboembolism episodes was also confirmed. However, this association has an unknown impact on medical practice until now.

## Author contributions

**Conceptualization:** Valter Romão de Souza Junior, Victor Moreira Valença de Lemos, Daniell de Siqueira Araújo Lafayette, Roberto Souza de Lemos, Luciana de Barros Correia Fontes, Glaydes Maria Torres de Lima, Cláudia Wanderley de Barros Correia, Ana Lucia Coutinho Domingues, Amanda Queiroz da Mota Silveira Aroucha, Igor Macedo de Oliveira, Gabriel Pinheiro Santos, Ana Maria Vanderlei, Vladmyr Moreira Valença de Lemos, João Sóstenes Peter, Juliana Oliveira Vieira, Aline Rayane Pereira Mariano, Turíbio Anacleto Gomes, Alice Marcelle de Souza Ferraz, José Henrique Souza do Amaral, ESDRAS MARQUES LINS, Marcos André Cavalcanti Bezerra, Paulo Sérgio Ramos de Araújo, Adriana Ferraz de Vasconcelos, Maria da Conceição de Barros Correia, Manuela Freire Hazin Costa.

**Data curation:** Valter Romão de Souza Junior, Victor Moreira Valença de Lemos, Daniell de Siqueira Araújo Lafayette, Roberto Souza de Lemos, Luciana de Barros Correia Fontes, Glaydes Maria Torres de Lima, Cláudia Wanderley de Barros Correia, Ana Lucia Coutinho Domingues, Amanda Queiroz da Mota Silveira Aroucha, Igor Macedo de Oliveira, Gabriel Pinheiro Santos, Ana Maria Vanderlei, Vladmyr Moreira Valença de Lemos, João Sóstenes Peter, Juliana Oliveira Vieira, Aline Rayane Pereira Mariano, Turíbio Anacleto Gomes, Alice Marcelle de Souza Ferraz, José Henrique Souza do Amaral, ESDRAS MARQUES LINS, Marcos André Cavalcanti Bezerra, Paulo Sérgio Ramos de Araújo, Adriana Ferraz de Vasconcelos, Maria da Conceição de Barros Correia, Manuela Freire Hazin Costa.

**Formal analysis:** Valter Romão de Souza Junior, Victor Moreira Valença de Lemos, Daniell de Siqueira Araújo Lafayette, Roberto Souza de Lemos, Luciana de Barros Correia Fontes, Glaydes Maria Torres de Lima, Cláudia Wanderley de Barros Correia, Ana Lucia Coutinho Domingues, Amanda Queiroz da Mota Silveira Aroucha, Igor Macedo de Oliveira, Gabriel Pinheiro Santos, Ana Maria Vanderlei, Vladmyr Moreira Valença de Lemos, João Sóstenes Peter, Juliana Oliveira Vieira, Aline Rayane Pereira Mariano, Turíbio Anacleto Gomes, Alice Marcelle de Souza Ferraz, José Henrique Souza do Amaral, ESDRAS MARQUES LINS, Marcos André Cavalcanti Bezerra, Paulo Sérgio Ramos de Araújo, Adriana Ferraz de Vasconcelos, Maria da Conceição de Barros Correia, Manuela Freire Hazin Costa.

**Funding acquisition:** Valter Romão de Souza Junior, Victor Moreira Valença de Lemos, Daniell de Siqueira Araújo Lafayette, Glaydes Maria Torres de Lima, Cláudia Wanderley de Barros Correia, Ana Lucia Coutinho Domingues, Amanda Queiroz da Mota Silveira Aroucha, Igor Macedo de Oliveira, Gabriel Pinheiro Santos, Ana Maria Vanderlei, Vladmyr Moreira Valença de Lemos, João Sóstenes Peter, Juliana Oliveira Vieira, Aline Rayane Pereira Mariano, Turíbio Anacleto Gomes, Alice Marcelle de Souza Ferraz, José Henrique Souza do Amaral, ESDRAS MARQUES LINS, Marcos André Cavalcanti Bezerra, Paulo Sérgio Ramos de Araújo, Adriana Ferraz de Vasconcelos.

**Investigation:** Valter Romão de Souza Junior, Victor Moreira Valença de Lemos, Daniell de Siqueira Araújo Lafayette, Roberto Souza de Lemos, Luciana de Barros Correia Fontes, Glaydes Maria Torres de Lima, Cláudia Wanderley de Barros Correia, Ana Lucia Coutinho Domingues, Amanda Queiroz da Mota Silveira Aroucha, Igor Macedo de Oliveira, Gabriel Pinheiro Santos, Ana Maria Vanderlei, Vladmyr Moreira Valença de Lemos, João Sóstenes Peter, Juliana Oliveira Vieira, Aline Rayane Pereira Mariano, Turíbio Anacleto Gomes, Alice Marcelle de Souza Ferraz, José Henrique Souza do Amaral, ESDRAS MARQUES LINS, Marcos André Cavalcanti Bezerra, Paulo Sérgio Ramos de Araújo, Adriana Ferraz de Vasconcelos, Maria da Conceição de Barros Correia, Manuela Freire Hazin Costa.

**Methodology:** Valter Romão de Souza Junior, Victor Moreira Valença de Lemos, Daniell de Siqueira Araújo Lafayette, Roberto Souza de Lemos, Luciana de Barros Correia Fontes, Glaydes Maria Torres de Lima, Cláudia Wanderley de Barros Correia, Ana Lucia Coutinho Domingues, Amanda Queiroz da Mota Silveira Aroucha, Igor Macedo de Oliveira, Gabriel Pinheiro Santos, Ana Maria Vanderlei, Vladmyr Moreira Valença de Lemos, João Sóstenes Peter, Juliana Oliveira Vieira, Aline Rayane Pereira Mariano, Turíbio Anacleto Gomes, Alice Marcelle de Souza Ferraz, José Henrique Souza do Amaral, ESDRAS MARQUES LINS, Marcos André Cavalcanti Bezerra, Paulo Sérgio Ramos de Araújo, Adriana Ferraz de Vasconcelos, Maria da Conceição de Barros Correia, Manuela Freire Hazin Costa.

**Project administration:** Valter Romão de Souza Junior, Victor Moreira Valença de Lemos, Daniell de Siqueira Araújo Lafayette, Roberto Souza de Lemos, Luciana de Barros Correia Fontes, Glaydes Maria Torres de Lima, Cláudia Wanderley de Barros Correia, Ana Lucia Coutinho Domingues, Amanda Queiroz da Mota Silveira Aroucha, Igor Macedo de Oliveira, Gabriel Pinheiro Santos, Ana Maria Vanderlei, Vladmyr Moreira Valença de Lemos, João Sóstenes Peter, Juliana Oliveira Vieira, Aline Rayane Pereira Mariano, Turíbio Anacleto Gomes, Alice Marcelle de Souza Ferraz, José Henrique Souza do Amaral, ESDRAS MARQUES LINS, Marcos André Cavalcanti Bezerra, Paulo Sérgio Ramos de Araújo, Adriana Ferraz de Vasconcelos, Maria da Conceição de Barros Correia, Manuela Freire Hazin Costa.

**Resources:** Valter Romão de Souza Junior, Victor Moreira Valença de Lemos, Daniell de Siqueira Araújo Lafayette, Roberto Souza de Lemos, Luciana de Barros Correia Fontes, Glaydes Maria Torres de Lima, Cláudia Wanderley de Barros Correia, Ana Lucia Coutinho Domingues, Amanda Queiroz da Mota Silveira Aroucha, Igor Macedo de Oliveira, Gabriel Pinheiro Santos, Ana Maria Vanderlei, Vladmyr Moreira Valença de Lemos, João Sóstenes Peter, Juliana Oliveira Vieira, Aline Rayane Pereira Mariano, Turíbio Anacleto Gomes, Alice Marcelle de Souza Ferraz, José Henrique Souza do Amaral, ESDRAS MARQUES LINS, Marcos André Cavalcanti Bezerra, Paulo Sérgio Ramos de Araújo, Adriana Ferraz de Vasconcelos, Maria da Conceição de Barros Correia, Manuela Freire Hazin Costa.

**Supervision:** Valter Romão de Souza Junior, Victor Moreira Valença de Lemos, Daniell de Siqueira Araújo Lafayette, Roberto Souza de Lemos, Luciana de Barros Correia Fontes, Glaydes Maria Torres de Lima, Cláudia Wanderley de Barros Correia, Ana Lucia Coutinho Domingues, Amanda Queiroz da Mota Silveira Aroucha, Igor Macedo de Oliveira, Gabriel Pinheiro Santos, Ana Maria Vanderlei, Vladmyr Moreira Valença de Lemos, João Sóstenes Peter, Juliana Oliveira Vieira, Aline Rayane Pereira Mariano, Turíbio Anacleto Gomes, Alice Marcelle de Souza Ferraz, José Henrique Souza do Amaral, ESDRAS MARQUES LINS, Marcos André Cavalcanti Bezerra, Paulo Sérgio Ramos de Araújo, Adriana Ferraz de Vasconcelos, Maria da Conceição de Barros Correia, Manuela Freire Hazin Costa.

**Validation:** Valter Romão de Souza Junior, Victor Moreira Valença de Lemos, Daniell de Siqueira Araújo Lafayette, Roberto Souza de Lemos, Luciana de Barros Correia Fontes, Glaydes Maria Torres de Lima, Cláudia Wanderley de Barros Correia, Ana Lucia Coutinho Domingues, Amanda Queiroz da Mota Silveira Aroucha, Igor Macedo de Oliveira, Gabriel Pinheiro Santos, Ana Maria Vanderlei, Vladmyr Moreira Valença de Lemos, João Sóstenes Peter, Juliana Oliveira Vieira, Aline Rayane Pereira Mariano, Turíbio Anacleto Gomes, Alice Marcelle de Souza Ferraz, José Henrique Souza do Amaral, ESDRAS MARQUES LINS, Marcos André Cavalcanti Bezerra, Paulo Sérgio Ramos de Araújo, Adriana Ferraz de Vasconcelos, Maria da Conceição de Barros Correia, Manuela Freire Hazin Costa.

**Visualization:** Valter Romão de Souza Junior, Victor Moreira Valença de Lemos, Daniell de Siqueira Araújo Lafayette, Roberto Souza de Lemos, Luciana de Barros Correia Fontes, Glaydes Maria Torres de Lima, Cláudia Wanderley de Barros Correia, Ana Lucia Coutinho Domingues, Amanda Queiroz da Mota Silveira Aroucha, Igor Macedo de Oliveira, Gabriel Pinheiro Santos, Ana Maria Vanderlei, Vladmyr Moreira Valença de Lemos, João Sóstenes Peter, Juliana Oliveira Vieira, Aline Rayane Pereira Mariano, Turíbio Anacleto Gomes, Alice Marcelle de Souza Ferraz, José Henrique Souza do Amaral, ESDRAS MARQUES LINS, Marcos André Cavalcanti Bezerra, Paulo Sérgio Ramos de Araújo, Adriana Ferraz de Vasconcelos, Maria da Conceição de Barros Correia, Manuela Freire Hazin Costa.

**Writing – original draft:** Valter Romão de Souza Junior, Victor Moreira Valença de Lemos, Daniell de Siqueira Araújo Lafayette, Roberto Souza de Lemos, Luciana de Barros Correia Fontes, Glaydes Maria Torres de Lima, Cláudia Wanderley de Barros Correia, Ana Lucia Coutinho Domingues, Amanda Queiroz da Mota Silveira Aroucha, Igor Macedo de Oliveira, Gabriel Pinheiro Santos, Ana Maria Vanderlei, Vladmyr Moreira Valença de Lemos, João Sóstenes Peter, Juliana Oliveira Vieira, Aline Rayane Pereira Mariano, Turíbio Anacleto Gomes, Alice Marcelle de Souza Ferraz, José Henrique Souza do Amaral, ESDRAS MARQUES LINS, Marcos André Cavalcanti Bezerra, Paulo Sérgio Ramos de Araújo, Adriana Ferraz de Vasconcelos, Maria da Conceição de Barros Correia, Manuela Freire Hazin Costa.

**Writing – review & editing:** Valter Romão de Souza Junior, Victor Moreira Valença de Lemos, Daniell de Siqueira Araújo Lafayette, Roberto Souza de Lemos, Luciana de Barros Correia Fontes, Glaydes Maria Torres de Lima, Cláudia Wanderley de Barros Correia, Ana Lucia Coutinho Domingues, Amanda Queiroz da Mota Silveira Aroucha, Igor Macedo de Oliveira, Gabriel Pinheiro Santos, Ana Maria Vanderlei, Vladmyr Moreira Valença de Lemos, João Sóstenes Peter, Juliana Oliveira Vieira, Aline Rayane Pereira Mariano, Turíbio Anacleto Gomes, Alice Marcelle de Souza Ferraz, José Henrique Souza do Amaral, ESDRAS MARQUES LINS, Marcos André Cavalcanti Bezerra, Paulo Sérgio Ramos de Araújo, Adriana Ferraz de Vasconcelos, Maria da Conceição de Barros Correia, Manuela Freire Hazin Costa.
